# Differential gene expression profiles of gastric cancer cells established from primary tumour and malignant ascites

**DOI:** 10.1038/sj.bjc.6600580

**Published:** 2002-11-04

**Authors:** C Sakakura, A Hagiwara, M Nakanishi, K Shimomura, T Takagi, R Yasuoka, Y Fujita, T Abe, Y Ichikawa, S Takahashi, T Ishikawa, I Nishizuka, T Morita, H Shimada, Y Okazaki, Y Hayashizaki, H Yamagishi

**Affiliations:** Department of Digestive Surgery, Kyoto Prefectural University of Medicine, Kamigyo-ku, Kawaramachi-dori, Kyoto 602-8566, Japan; Department of Hygiene, Kyoto Prefectural University of Medicine, Kamigyo-ku, Kawaramachi-dori, Kyoto 602-8566, Japan; Department of Surgery-2, Yokohama City University, School of Medicine, 3-9 Fukuura Kanazawa-ku, 236-0004, Yokohama, Japan; Genomic Sciences Center, RIKEN, Yokohama Institute, 1-7-22 Suehiro-cho, Tsurumi-district, Yokohama, 230-0045, Japan

**Keywords:** peritoneal dissemination, gastric cancer, cDNA microarray

## Abstract

Advanced gastric cancer is often accompanied by metastasis to the peritoneum, resulting in a high mortality rate. Mechanisms involved in gastric cancer metastasis have not been fully clarified because metastasis involves multiple steps and requires a combination of altered expressions of many different genes. Thus, independent analysis of any single gene would be insufficient to understand all of the aspects of gastric cancer peritoneal dissemination. In this study, we performed a global analysis of the differential gene expression of a gastric cancer cell line established from a primary main tumour (SNU-1) and of other cell lines established from the metastasis to the peritoneal cavity (SNU-5, SNU-16, SNU-620, KATO-III and GT3TKB). The application of a high-density cDNA microarray method made it possible to analyse the expression of approximately 21 168 genes. Our examinations of SNU-5, SNU-16, SNU-620, KATO-III and GT3TKB showed that 24 genes were up-regulated and 17 genes down-regulated besides expression sequence tags. The analysis revealed the following altered expression such as: (a) up-regulation of CD44 (cell adhesion), keratins 7, 8, and 14 (epitherial marker), aldehyde dehydrogenase (drug metabolism), CD9 and IP3 receptor type3 (signal transduction); (b) down-regulation of IL2 receptor γ, IL4-Stat (immune response), p27 (cell cycle) and integrin β4 (adhesion) in gastric cancer cells from malignant ascites. We then analysed eight gastric cancer cell lines with Northern blot and observed preferential up-regulation and down-regulation of these selected genes in cells prone to peritoneal dissemination. Reverse transcriptase–polymerase chain reaction confirmed that several genes selected by DNA microarray were also overexpressed in clinical samples of malignant ascites. It is therefore considered that these genes may be related to the peritoneal dissemination of gastric cancers. The results of this global gene expression analysis of gastric cancer cells with peritoneal dissemination, promise to provide a new insight into the study of human gastric cancer peritoneal dissemination.

*British Journal of Cancer* (2002) **87**, 1153–1161. doi:10.1038/sj.bjc.6600580 www.bjcancer.com

© 2002 Cancer Research UK

## 

Gastric cancer is the most frequent malignancy of the gastrointestinal tract among Japanese and certain South-East Asian populations and the second most common cause of cancer-related death in the world (Parkin *et al*, 1999). The prognosis of gastric cancer has been improving owing to progress in diagnostic techniques and treatment methods for gastric cancer, but peritoneal dissemination is the main cause of recurrence after curative resection of advanced cancer. The prognosis of gastric cancer which has invaded as far as the gastric serosa is still poor with a 5-year survival of less than 35% (Yamazaki *et al*, 1989; Shimada and Ajani, 1999). Among these malignant characteristics of gastric cancer cells, metastasis to the peritoneum is an especially complex phenomenon, which requires the involvement of many different genes in multiple steps for tumour cells. Although many aspects of gastric cancer metastasis await further clarification, adhesion molecules, apoptosis-related genes, and others have been reported to play an important role in peritoneal dissemination of gastric cancers (Tahara *et al*, 1996; Yawata *et al*, 1998; Tahara, 2000). It is well known that changes in the expression of these genes enhance cell escape from the primary tumour and resistance to apoptosis, but details of the mechanism involved remain unclear.

Most gastric cancer cell lines have been established from liver metastases, lymph node metastases, or malignant ascites, while few have been established from primary lesions. To investigate the mechanism of gastric cancer peritoneal dissemination, Park *et al* (1990, 1994) established several gastric cancer cell lines, SNU-1, SNU-5, SNU-16, and SNU-620 cells, SNU-1 from primary tumour, and others from malignant ascites, which have a high potential for peritoneal dissemination. SNU cell lines have been studied both extensively and intensively. cMET is amplified in SNU-16, overexpression of TGF-β type II receptor, CEA, CA19-9, and c-erbB 2 has been confirmed in SNU-1, SNU-5 and SNU-16, KATO-III cells have K-sam amplification (Katoh *et al*, 1992), and GT3TKB reduced expression of E-cadherin (Tamura *et al*, 1996). But their common changes of gene expression have not yet been clarified.

Coordination of multiple genes is involved in metastasis, whereas only one gene or a few genes have been the subject of most previous reports on metastasis. Moreover, differences in metastatic potential are expected to be due to a combination of differently expressed genes. In this context, gene expression analysis of gastric cancer cells with different metastatic potentials in terms of grade and target is extremely relevant for the clarification of the mechanism of gastric cancer peritoneal dissemination. For this study, we performed globally analysed expression profiles of approximately 21 000 genes in SNU-1, SNU-5, SNU-16, SNU-620, KATO-III, and GT3TKB cells by using a cDNA microarray. By combining this analysis with subsequent confirmation of altered expressions of selected genes by Northern blot analysis or RT–PCR, differently expressed genes among these cells could be positively identified. Although further functional analysis is necessary, our results can be expected to prove new insights into the mechanism of gastric cancer metastasis.

## MATERIALS AND METHODS

### Cell culture and RNA preparation

Gastric cancer cell lines SNU-1, SNU-5, SNU-16, SNU-620 cells were established previously by Park *et al* (1990, 1994, 1997). KATO-III, GT3TKB and MKN 7 were purchased from Riken Cell Bank (Tsukuba, Japan) (Hojo, 1977; Sekiguchi *et al*, 1978; Tamura *et al*, 1996). NUGC-3 cells were purchased from Health Science Research Resources Bank (Osaka, Japan). GT3TKB were maintained at 37°C in a humidified atmosphere of 5% CO_2_ in high-glucose DMEM (Sigma, St. Louis, MO, USA), whereas MKN7 and NUGC-3 were maintained in RPMI 1640 (Sigma). Both media were supplemented with 10% foetal bovine serum, penicillin, and streptomycin. When they reached 80–90% confluence, cells were washed with ice-cold PBS and homogenised immediately. mRNA extracted from each cell line was extracted by FAST track kit Ver.2 (Invitrogen) according to the manufacturer's instructions. Characteristics of these gastric cancer cell lines (origin, histology, potency of intraperitoneal transplantation) are shown in Table 1

**Table 1:**
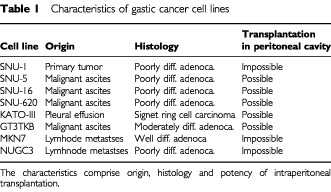
Characteristics of gastic cancer cell lines

.

### Experimental model in nude mouse

Four-week-old female BALB/c nu/nu mice (Clea Japan, Inc., Osaka, Japan) were inoculated intraperitoneally with 5×10^6^ gastric cancer cells in 0.5 ml PBS. Four weeks later, the presence of disseminated foci or ascites was determined. All animal experiments were conducted in accordance with institutional guidelines for animal welfare.

### Preparation of probe

One microgram of mRNA extracted from each gastric cancer cell line was labelled by incorporating Cy3 during random-primed reverse transcription. cDNA derived from the reference pool, which we labelled with Cy5, was used as the expression reference. We combined mRNA from the following cells in equal quantities to make the reference pool: HL60 (acute myeloid leukaemia) and K562 (chronic myeloid leukaemia); NCI-H226 (non-small cell lung); COLO205 (colon); SNB-19 (central nervous system); LOX-IMVI (melanoma); OVCAR-3, OVCAR-4 (ovary); CAKI-1 (kidney); PC-3 (prostate); and MCF7 and HS 578T (breast). Twelve highly diverse cell lines were selected on the basis of the results of previous studies (Ross *et al*, 2000; Scherf *et al*, 2000). Cell lines were selected from various organs to ensure that the reference RNA contained as many different kinds of gene transcripts as possible.

The labelling was carried out at 42°C for 1 h in a total volume of 30 μl containing 400 units of SuperScript II (GIBCO/BRL); 0.1 mM Cy3-dUTP (or Cy5-dUTP); 0.5 mM each dATP, dCTP, and dGTP; 0.2 mM dTTP, 10 mM DTT, 6 μl of 5×first-strand buffer, and 6 μg of random primers. To remove unincorporated nucleotide, labelled cDNA was mixed with 500 μl of binding buffer (5 M guanidine thiocyanate per 10 mM Tris·HCl, pH 7.0 per 0.1 mM EDTA containing 0.03% gelatin and 2 ng μl^−1^ tRNA) and 50 μl of silica matrix buffer (10% matrix/3.5 M guanidine hydrochloride/20% glycerol/0.1 mM EDTA/200 mM NaOAc, pH 4.8–5.0), transferred to a GFX column (Amersham Pharmacia), and centrifuged at 15 000 r.p.m. in a Sorvall centrifuge (RC-3B plus; H6000A/HBB6 rotor) for 30 s. The flow-through was discarded, and the column was washed with 500 μl of wash buffer. The adsorbed probe was eluted into a final volume of 17 μl of distilled water. This labelled probe was mixed with blocking solution containing 3 μl of 10 μg μl^−1^ oligo(dA), 3 μl of 20 μg μl^−1^ yeast tRNA, 1 μl of 20 μg μl^−1^ mouse Cot1 DNA, 5.1 μl of 20×SSC, and 0.9 μl 10% SDS (Miki *et al*, 2001).

### Array hybridisation and data analysis

The RIKEN human cDNA that comprised the target was hybridised in a final volume of 30 μl; the entire array consists of three multiblocks, and each multiblock required 10 μl of hybridisation solution. Before hybridisation, probe aliquots were heated at 95°C for 1 min and cooled at room temperature. Coverslips were hybridised overnight at 65°C in a Hybricasette (obtained from ArrayIt.com). After hybridisation, slides were washed in 2×SSC/0.1% SDS until the coverslips dropped off, and the slides were then transferred into 1×SSC, shaken gently for 2 min, and rinsed with 0.1×SSC for 2 min. After washing, slides were spun at 800 r.p.m. in a Sorvall centrifuge (RC-3B plus; H6000A/HBB6 rotor). These slides were scanned on a ScanArray 5000 confocal laser scanner, and the images were analysed by using IMAGENE (BioDiscovery; Los Angeles, CA, USA).

### Analysis of the data

To improve the accuracy of the data, we did the experiment twice, labelling the same RNA template in two separate reactions. Data were normalised to the reference standard by subtracting (in log space) the median observed value if it were other than zero. We used only data points that were reproducible. To this end, we developed a filtering program, PRIM (Kaota *et al*, 2001). Briefly, this program (i) deletes the results with ‘flags’ added manually to corrupted spots; (ii) eliminates spots with signal intensities less than the mean + 3×standard deviation of the background signal intensity in either Cy3 or Cy5; and (iii) eliminates spots located outside the least-mean-squares line ±2×standard deviation. After the filtering was finished, we compared the results of the two experiments by calculating a Pearson's correlation coefficient. If the coefficient was equal to or greater than 0.7, we used the data in subsequent analyses. If not, we repeated the labelling, hybridisation, and scanning up to six times. In this way, we could generate high-quality data for most tissues. Preceeding the clustering, ratio values from duplicate experiments were averaged, log-transformed (base 2), and stored in a table. We applied hierarchical clustering to both axes, using the weighted pair-group method with a centroid average as implemented by the program CLUSTER (Eisen *et al*, 1998). The results were analysed by using TREEVIEW (Eisen *et al*, 1998).

### Northern blot analysis

Northern blot was performed as we described previously (Sakakura *et al*, 1994, 1996). In brief, total cellular RNA was prepared by the guanidine isothiocyanate-phenol-chloroform procedure. Selection of poly(A)^+^ RNA was performed by an oligo dT column, then fractionated on 1% agarose/2.2 M formaldehyde gels. Probes were labelled with ^32^P by random priming. Each blot was hybridised with probes for each selected gene and β-actin. We analysed signals with a BAS 2000 image analyser and calculated the degree of overexpression compared to control.

### Clinical samples of malignant ascites and peritoneal washes of benign disease

The study population is consist of 10 patients of gastric cancer with malignant ascites (with positive cytology results) and 10 patients with benign disease including cholecystolithiasis and leiomyoma of the stomach, undergoning surgery at Kyoto Prefectural University of Medicine. Ascites fluid was collected from the Douglas cavity at laparotomy. In the absence of ascites, 50 ml of saline was introduced into the Douglas cavity at the beginning of the operation and aspirated after general stirring. These washes were centrifuged at 2000 r.p.m. for 10 min to collect intact cells, rinsed with PBS, dissolved in ISOGEN RNA extraction buffer (Nippon Gene, Tokyo), and stored at –80°C until use. All experiments with clinical samples were conducted under institutional guideline from the Ministry of Health and Welfare.

### Reverse transcriptase–polymerase chain reaction (RT–PCR)

cDNA was produced from total RNA by using a Superscript preamplification system (BRL, Bethesda, MD, USA) and following the procedures suggested by the manufacturer. RNA was heated to 65°C for 10 min in 14 μl of duethylpyrocarbonate-treated water containing 0.5 μg oligo (dT). Synthesis buffer (10×), 2 μl 10 mM dNTP mix, 2 μl 0.1 M DTT, and reverse transcriptase (Superscript RT; 200 U μl^−1^) were added to the sample. The resulting reaction mixture was incubated at 42°C for 50 min, and reaction was terminated by incubating the mixture at 95°C for 5 min.

The sequences of the three sets of primer used were: Inositol triphospate receptor (IP3R) (sense) 5′-CACGTGAAGTGGGCCATAAC-3′, (antisense) 5′-TCCGTCAGGAACTGGCAGAT-3′; Keratin 7 (sense) 5′-ACCATTAACCAGAGCCTGCT-3′, (antisense) 5′-TCATTCAGGGCATCCACCTT-3′; aldehyde dehydrogenase (ALDH) (sense) 5′-ATTGTGTTAGCTGATG CCGACTT-3′ (antisense) 5′-CACTGGCCCTGGTGGTAGAATA-3′. PCR was carried out in a reaction mixture (10 μl) containing 10 mM Tris-HCl, pH 8.3, 50 mM KCl, 200 μM dNTP, 1.5 mM MgCl_2_, 0.25 unit of Taq DNA polymerase (Perkin-Elmer Cetus, Branchburg, NJ, USA), 0.2 μM primers A and B, and 1 μl of template cDNA. Twenty-five rounds of amplification were performed in a thermocycler (MJ Research Inc., Watertown, MA, USA) at 94°C for 30 s, 58°C for 30 s, and 72°C for 30 s, with a final extension step at 72°C for 10 min. After PCR, 5 μl of the reaction mixture was electrophoresed on a 2% agarose gel.

## RESULTS

### Global gene expression analysis by cDNA microarray of gastric cancer cell lines from malignant ascites

**Figure 1:**
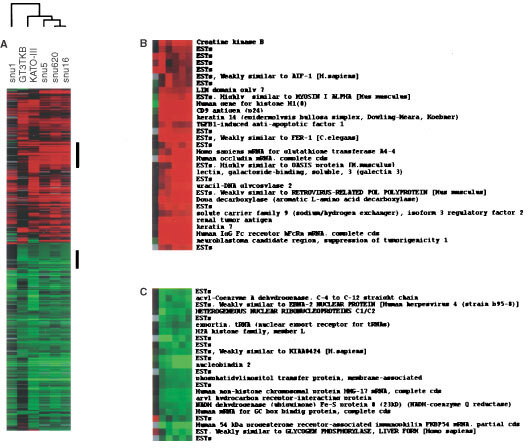
High-density cDNA microarray analysis. Validation in expression of selected genes from 21 68 clones in six gastric cancer cell lines. Data are represented in a matrix format: each row represents a single gene, and each column an experimental sample. In each sample, the ratio of the number of transcripts of each gene to the medium abundance of the gene's transcript among all the cell lines is represented by the colour of the corresponding cell in the matrix. Green square,transcript level below the median; black squares, transcript levels equal to the medium; red squares, transcript levels greater than the median. Colour saturation reflects the magnitude of the ratio relative to the median for each set of samples. (**A**) Cluster analysis of selected genes of six gastric cancer cell lines using the RIKEN cDNA microarray. Bars to the right identify the location of the inserts displayed in panels **B** and **C**. (**B**) Up-regulated genes in gastric cancer cells from malignant ascites. (**C**) Down-regulated genes in gastric cancer cells from malignant ascites

We performed a global analysis of gene expression of 21 168 genes in SNU-1, SNU-5, SNU-16, SNU-620, KATO-III and GT3TKB cells using a high-density cDNA microarray and compared the gene expression profiles of SNU-5, SNU-16, SNU-620, KATO-III and GT3TKB cells with that of SNU-1. To eliminate data with low reliability, genes whose expression was regarded as absent in both cell lines as a result of software analysis were excluded. The results of the analysis by TREEVIEW are shown in Figure 1

. Among the 11 680 to 15 151 genes (known genes and ESTs) expressed, 44 genes showed a differential expression more than double that of SNU-1 and 30 more than half that of other cell lines. To verify these findings, we performed Northern blot to analyse all these genes that showed greater than two-fold difference in terms of cDNA array. When more than two-fold changes in expression level by Northern blot analysis were considered significant, consistency with cDNA microarray analysis were 55% (41 of 74), whereas no significant change was seen in 27% (20 of 74), and discordant results were obtained in 18% (13 of 74). Genes identified both analyses are listed in Tables 2

**Table 2:**
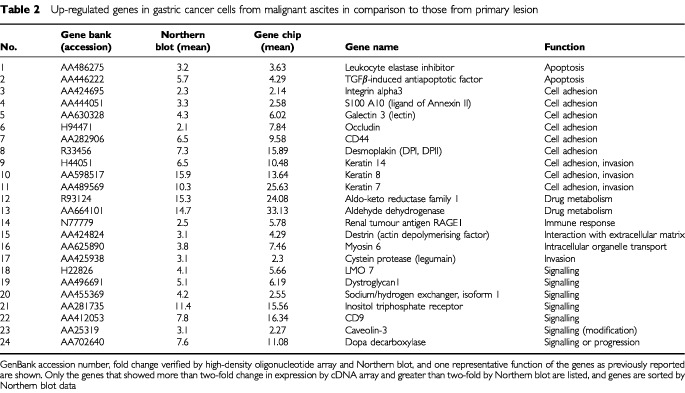
Up-regulated genes in gastric cancer cells from malignant ascites in comparison to those from primary lesion

and 3

**Table 3:**
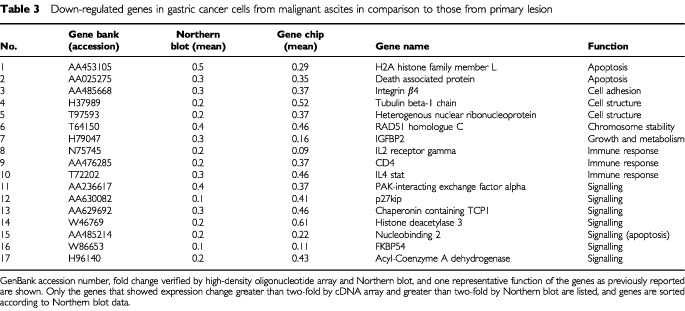
Down-regulated genes in gastric cancer cells from malignant ascites in comparison to those from primary lesion

**Figure 2:**
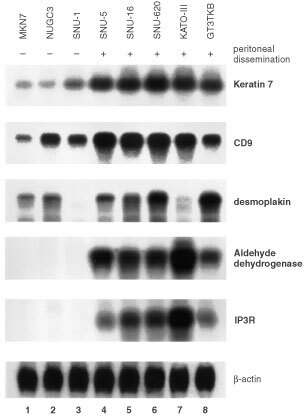
Northern blot analysis of differently expressed genes in eight gastric cancer cells. Up-regulated genes in gastric cancer cells from malignant ascites in comparison to primary lesion

. Typical data are shown in Figures 2

and 3

**Figure 3:**
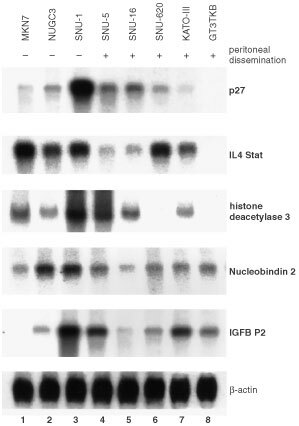
Northern blot analysis of differently expressed genes in eight gastric cancer cells. Down-regulated genes in gastric cancer cells from malignant ascites other than those from primary lesion

.

### Expression of selected genes in eight gastric cancer cell lines

Because the peritoneal dissemination potentials of other gastric cancer cell lines have been reported by several previous studies, we also investigated the expression of the differential gene expression of SNU-1 and other gastric cancer cells by Northern blot analysis. We performed this analysis on another two gastric cancer cell lines in addition to the six cell lines analysed by cDNA microarray, to find any correlation between these genes and the peritoneal dissemination potential of gastric cancer cells (Figures 2 and 3). KATO-III, SNU-5, SNU-16, and SNU-620 are often used as a peritoneal dissemination model in nude mice because of their reportedly high potential for peritoneal dissemination. Although there are no reports on this potential of GT3TKB in nude mice, we confirmed it in an animal experiment, in which NUGC-3 and MKN7 did not generate a peritoneal metastasis even 4 weeks after inoculation. These findings indicated that these cells can be readily classified into three groups: (a) cells with a high potential for peritoneal dissemination (SNU-5, SNU-16, and SNU-620, KATO-III and GT3TKB), (b) main tumour-derived cell line SNU-1 with no potential for peritoneal dissemination and (c) MKN7 and NUGC-3 with low potential or no potential for peritoneal dissemination.

In addition to MKN7 and NUGC3, most genes, CD44 and Keratin family genes are strongly expressed in gastric cancer cells of peritoneal dissemination. Several genes, such as aldehyde dehydrogenase and IP3R, showed a high expression level only in cells with a high potential for such dissemination and a low expression level in all of the cells with a low potential for peritoneal dissemination. These changes in expression may be specific for gastric cancer peritoneal dissemination. Most down-regulated genes, such as p27 are also repressed in all other gastric cancer cell lines, while some of the listed genes, such as IL4 Stat, are more intensively down-regulated in gastric cancer cells of peritoneal dissemination.

### Experimental model in nude mouse

Intraperitoneal inoculation of KATO-III or GT3TKB cells resulted in nodules in the peritoneal cavity, while the other cell lines, SNU-5, SNU-16, and SNU-620, formed nodules and malignant ascites. In contrast, no disseminated foci could be detected after intraperitoneal inoculation of either MKN7 or NUGC3. Potency of intraperitoneal transplantation in these cancer cells is shown in Table 1.

### RT–PCR

**Figure 4:**
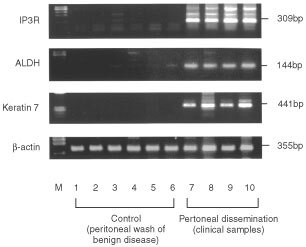
Expression of selected genes in clinical samples of peritoneal dissemination. Representative RT–PCR results of six peritoneal washes from patients with benign disease (cytology negative) and from four gastric cancer patients with malignant ascites (cytology positive). Arrows indicate the specific bands of each gene

A specific PCR fragment was successfully amplified by RT–PCR for each set of primers with the expected size. Direct sequencing confirmed that these bands could be identified with each of the amplified sequences. The specific band was detected in all ten samples of malignant ascites tested with each of three different target genes. In contrast, none of the 10 patients with benign disease was positive, although shallow bands were detected in some of them. Typical data are shown in Figure 4

. These results suggest that genes selected by DNA microarray were overexpressed in the gastric cancer cell lines established from the metastases to the peritoneal cavity as well as in the clinical samples of malignant ascites.

## DISCUSSION

The introduction of novel improved array technologies has resulted in new possibilities for studying the molecular mechanisms of cancer metastasis. In our study, the relative mRNA levels of genes expressed in the cell lines examined were identified by using cDNA microarrays. The array analysis suggested that the genes shown in Table 2 were up-regulated and those in Table 3 were significantly down-regulated. With regard to peritoneal dissemination, malignant characteristics of cancer cells such as reduced intercellular adhesion, increased cell-to-matrix adhesion, and resistance to apoptosis are considered to be important features. Several of the 41 genes that showed different expression levels in SNU-1 and other cell lines different from those of malignant ascites have been previously reported to be related to these features. The possible involvement of other genes listed in Tables 2 and 3 to peritoneal dissemination of gastric cancers has not yet been reported. Other expression sequence tags (ESTs) can be expected to be involved in peritoneal dissemination, but the precise function of each gene remains unclear, so that further study would be necessary to clarify it.

Previous reports have shown gene expression profiles in schirrous gastric cancer cell lines as well as xenografts of gastric cancers in nude mice (El-Rifai *et al*, 2001; Hippo *et al*, 2001). As we screened more than 21 000 genes, we could select more novel genes that have not reported previously in gastric cancers. Selected genes of Tables 2 and 3 were singled out according to their function and are discussed in relation to previous reports.

### Genes related to signalling (growth and metabolism)

Although growth factors, their ligands and down-stream molecules are frequenly overexpressed in cancer cells, to the best of our knowledge, several genes related to signalling as listed in Table 2, have not been reported previously. The extracellular domain of CD9 binds the epidermal growth factor (EGF) and enhances the function of EGF signalling (Nakamura *et al*, 2000; Hashida *et al*, 2002). Dopa decarboxylase (DDC) is responsible for the synthesis of the key neurotransmitters dopamine and serotonin, and is frequently expressed in neuroblastoma and small cell carcinoma of the lung (North and Du, 1998; Gilbert *et al*, 1999). Sodium/hydrogen exchanger isoform 1, is activated more in malignant gliomas than nontransformed astrocytes, and malignant gliomas display altered pH regulation (Lagana *et al*, 2000; Mclean *et al*, 2000). LMO7 in gastric cancer has not been dealt with in any reports, but another isoform, LMO4 (a transcriptional regulator) inhibits differentiation of mammary epithelial cells *in vitro* and is overexpressed in breast cancers (Putilina *et al*, 1998; Kurihara *et al*, 2002). Inositol 1, 4, 5-triphospate receptor type 3 (IP(3)R3) is frequently expressed in secretion cells and known to be related to the IP3 signalling pathway, Ca^2+^ influx, enzyme secretion and cell motility (Broad *et al*, 2001; Lee and Laychock, 2001), but its role in peritoneal dissemination is still unclear. Aldoketoreductase and aldehyde dehydrogenase (ALDH) belong to a family of several isoenzymes important in cell defence against both exogenous and endogenous aldehydes (Deichmann *et al*, 1999; Canuto *et al*, 2001). Compared with expression of ALDH in normal hepatocytes, several changes were observed in rat hepatoma cells (Canuto *et al*, 2001). PAK-interacting exchange factor (PIX) has been reported to mediate the recruitment of PAK into focal adhesions and thus to create a feedback loop that stimulates PAK and other molecules (Wang *et al*, 2001). Down-regulation of histon deacetylase 3 has also been detected, as has inactivation of many tumour suppressor genes and cell cycle regulators by methylation (Ozawa *et al*, 2001). Histon deacetylase 3 was down-regulated in tumour cell lines and may be related to the suppression of tumour suppressor genes or cell cycle regulators. Prenatal diagnosis of multiple acyl-CoA dehydrogenase deficiency associated with elevated alpha-fetoprotein and cystic renal change has been established (Chisholm *et al*, 2001). But the precise roles of these genes in metastasis have not been identified, however, so that further study is necessary.

### Genes related to cell adhesion and motility

Several genes related to cell adhesion, invasion, adhesion to extracellular matrix, and growth factors were abundantly over-expressed in our cell lines derived from malignant ascites. CD44 (Hsieh *et al*, 1999), keratins 7 and 8 (Martens *et al*, 1999) are overexpressed in several different kinds of cancers. Galectin 4 has been found to be overexpressed in breast cancers and schirrous gastric cancer cell lines (Hippo *et al*, 2001; Moon *et al*, 2001). The involvement of integrin alpha 3 and beta 4 have also been reported in cancer metastasis (Ishii *et al*, 2000; Chu *et al*, 2001; Galbiati *et al*, 2001). However, to our knowledge, no other reports related to galectin 3, S100A10, desmoplakin, occludin, keratins 8, 14, myosin VI, tubulin beta 4 and calveolin-3 in gastric cancer have been published. Calcium binding protein S100A10 is reported to be overexpressed in renal cell carcinoma (Teratani *et al*, 2002) and may exert its effect on metastasis formation by stimulating the motility and invasive properties of gastric cancer cells. Calveolin-3, a muscle-specific caveolin-related protein is the principal structural protein of caveolar membrane domains, which appear to be important for signal transduction in skeletal muscle and in the heart (Razani *et al*, 2000). Keratin family genes, such as Keratins 7, 8, and 14, have different specificity, and some of them are reportedly expressed in cancer cells to facilitate the latter's attachment to the peritoneum, and to promote liver metastasis of some cancers. They are also known to be involved in cell migration, cell invasiveness, plasminogen activity and drug and radiation resistance (Martens *et al*, 1999). Myosin VI is expressed in a variety of cell types and is thought to play a role in membrane trafficking and and endocytosis (Rock *et al*, 2001; Yoshimura *et al*, 2001). Tubulin beta 4 is downregulated in hepatoma cells than in normal liver cells (Yu *et al*, 2000). Although desmoplakin and dystroglycan are commonly down-regulated in clinical cancer specimens, they were overexpressed in our study (Davies *et al*, 1999; Henry *et al*, 2001). Their expression may be up-regulated again when cancer cells are disseminated on the methelium. Whether these genes are actually involved in peritoneal dissemination is a matter to be further investigated.

### Genes related to immunity

Down-regulation of CD4, IL2 receptor gamma and IL4–Stat genes is extremely intriguing. CD4 and CD8 are co-receptor molecules on lymphocytes and generally perform a helper or cytotoxic function. Although loss of CD4 expression on lymphocytes is known to correlate with the cytotoxic function, the role of CD4 expression in cancer cells remains unclear. Down-regulation of IL4 Stat, which is an important regulator of signalling from IL13 through the IL4R–STAT6 pathway, is necessary for down-regulation of tumour immuno-surveillance from NKT cells. It is reported that both IL4 and IL13 share signalling events in human colon carcinoma cell lines (HT29 and WiDr), and that both IL13 and IL4 induce phosphorylation of IL4 STAT (STAT6) (Murata *et al*, 1996; Terabe *et al*, 2000). The leukocyte elastase inhibitor is one of the possible target genes of PTEN (Hong *et al*, 2000). Up-regulation of leukocyte elastase inhibitor is thought to protect cancer cells from damage by immune cells, so that weakness of the IL13–IL4 Stat signalling pathway would allow cancer cells to escape from the immune response of the host. FKBP54 is known as a novel immunophilin (Milad *et al*, 1995; Zhu *et al*, 2001).

### Genes related to apoptosis and cell cycle

Histone H2A is related to chromatin condensation in TGFβ1-induced apoptosis (Marushige and Marushige, 1995), while heterogenous nuclear ribonucleoprotein A1 seems to be related to the potensity to develop metastases (Ghigna *et al*, 1998). Using COXs as bait in the yeast two-hybrid system, we identified autoimmunity and apoptosis-associated nucleobindin (Nuc) as a protein that specifically interacts with both isoenzymes (Ballif *et al*, 1996). It is known a reduced expression of CDK inhibitor p27 produces a malignant phenotype of cancer cells (Porter *et al*, 1997; Xiangming *et al*, 2000). Post-transcriptional regulation of p27 has been reported. Although p27 down-regulation would not be specific to peritoneal dissemination, p27 may be involved in cancer progression such as peritoneal dissemination.

Gene expression changes in adhesion molecules, signalling molecules, growth factor receptors, drug metabolism and immune response are likely to contribute to the complexicity of gastric cancer peritoneal dissemination. Overexpression or down-regulation of these genes in highly metastatic cells, as we have shown in this study, is in good concordance with the findings of previous reports (El-Rifai *et al*, 2001; Hippo *et al*, 2001) and indicates that gene expression changes in these cells accurately reflects some metastatic characteristics. For example, CD44 and galectin 3 (Hsieh *et al*, 1999; Moon *et al*, 2001), genes relevant for cancer metastasis, were up-regulated in peritoneal metastasis. Integrin β_4_ has recently been reported to be a suppressor of peritoneal dissemination of gastric cancer (Ishii *et al*, 2000). But our results and those of previous reports are somewhat different, suggesting that histological differences in samples used for these studies is responsible for the difference in gene expression.

Although the initial discrepancy between the data obtained in this study with high-density cDNA microarray and those obtained with Northern blot was not negligible, the final data presented here were verified by two different methods and are considered be highly reliable. cDNA microarray analysis greatly facilitated clarification of a major aspect of gastric cancer metastasis by identifying the global changes in gene expression. In this study, however, these genes showed different expression patterns among the eight gastric cancer cell lines, and some genes showed apparent correlation, while others did not, with their peritoneal dissemination potential. These results clearly show that a complex *in vivo* phenomenon such as peritoneal dissemination can not be explained by altered expression of a single gene. Thus, the relevance of exploring the global gene expression profile by using a comprehensive procedure is obvious.

In conclusion, a combination of gene expression changes in these genes leads to peritoneal dissemination of gastric cancers. This implies that autocrine production of these growth factors and their receptors by cancer cells may strongly promote invasion and metastasis. Whether these genes are actually involved in peritoneal dissemination, however, is a matter to be further investigated. A comparison between gene expression profiles of highly metastatic cell lines may identify coordinate genes that function as biological markers of peritoneal dissemination. Most of the up-regulated genes listed in Table 2 are overexpressed in most gastric cancer cell lines, but a few genes are likely to be strongly expressed in gastric cancer cells of peritoneal dissemination. Our preliminary experiments indicate that at least several of the genes listed in Table 2 were also frequently overexpressed in clinical samples of peritoneal dissemination. Identification of such genes could then lead to new therapeutic modalities as well as therapeutic targets.
